# Supplementary material for: Direct measurement of the Criegee intermediate CH2OO in ozonolysis of ethene
Source: Nat Commun. 2025 Jul 15;16:6515. doi: 10.1038/s41467-025-61739-5 (PMC12264038; doi:10.1038/s41467-025-61739-5)
Supplement: Supplementary file 1 — Supplementary information [file 41467_2025_61739_MOESM1_ESM.docx]

**Supplementary information for**

Direct Measurement of the Criegee Intermediate CH_2_OO in Ozonolysis of Ethene

**Authors:** Mixtli Campos-Pineda^1,2†^, Lei Yang^1†^, and Jingsong Zhang^1,3✉^

**Affiliations:**

^1^Department of Chemistry, University of California, Riverside, 92521, USA

^2^Present address: Centre for Research into Atmospheric Chemistry, University College Cork, T12 YN60, Ireland

^3^Air Pollution Research Center, University of California, Riverside, 92521, USA

^†^These authors contributed equally to this work

^✉^Corresponding author. Email: jingsong.zhang@ucr.edu

Contents:

Supplementary Figure 1. CH_2_OO concentration profile along the reactor

Supplementary Figure 2. HCHO spectra features at 374 – 392 nm

Supplementary Figure 3. Spectra with SO_2_ scavenger at 378 – 387 nm

Supplementary Figure 4. Change of spectra under varied conditions

Supplementary Figure 5. CH_2_OO loss pathway contributions

Supplementary Figure 6. Uncertainty analysis

Supplementary Figure 7. Yield of stabilized CH_2_OO at low pressure

Supplementary Figure 8. Concentrations of HCHO under varied conditions

Supplementary Figure 9. Kinetic study on CH_2_OO + SO_2_

Supplementary Figure 10. Experimental setup

Supplementary Figure 11. CH_2_OO reference spectra

Supplementary Table 1. Flow parameters of the reactor

Supplementary Table 2. Full simulation reaction network

Supplementary Table 3. Pseudo-first order reaction rates

Supplementary references


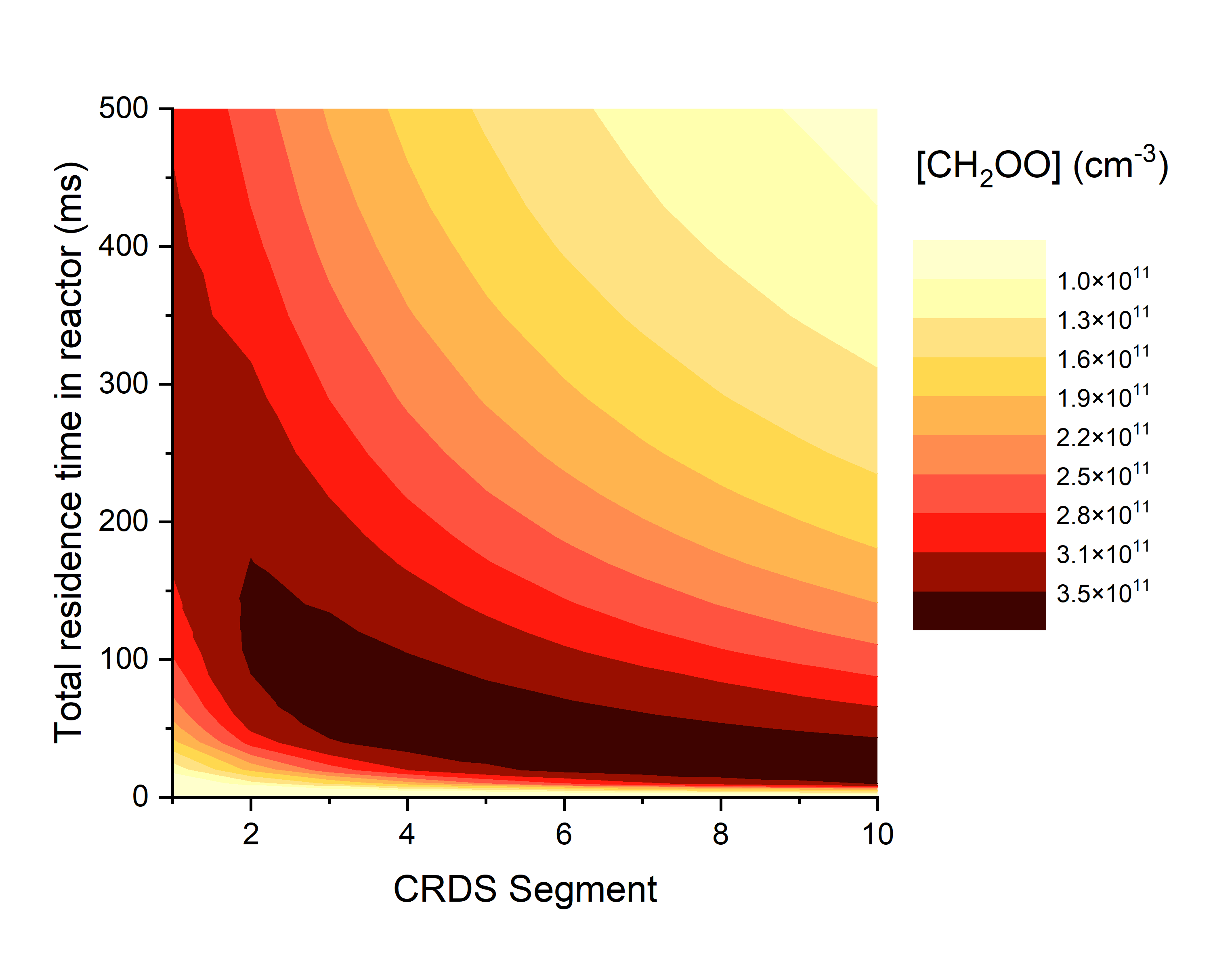


**Supplementary Figure 1.** **The concentration profile of CH_2_OO along the flow reactor** at different total residence times (0 – 500 ms), obtained from kinetic simulation with [ethene]_i_=1×10^17^ cm^−3^, [O_3_]_i_=1.8×10^15^ cm^−3^. The segments of the flow cell were modeled as CSTRs and the whole reactor was modeled as CSTRs in series. Source data are provided as a Source Data file.

**Supplementary Figure 2.** **Measured absorption coefficient in the ethene ozonolysis reaction at long residence time** (> 3 s) and comparison to the HCHO reference spectra^1^. The spectra correspond to ã^3^A_2_ ← X̃^1^A_1_ transitions of formaldehyde^2^. HCHO concentrations from fitting the two spectra are ~8×10^15^ cm^−3^. Broad absorption represents unidentified absorption from other species. Source data are provided as a Source Data file.

**Supplementary Figure 3.** **Absorption spectra measured during ozonolysis of ethene with the presence of SO_2_ scavenger** (~9×10^14^ cm^−3^). The CH_2_OO concentration decreased by >95% after the addition of the scavenger (from 2.62×10^11^ cm^−3^ without SO_2_ to ~9×10^9^ cm^−3^ with SO_2_). Reference spectra of SO_2_ (Vandaele et al.^3^) were obtained from the MPI-Mainz UV/VIS Spectral Atlas^4^. Source data are provided as a Source Data file.

**
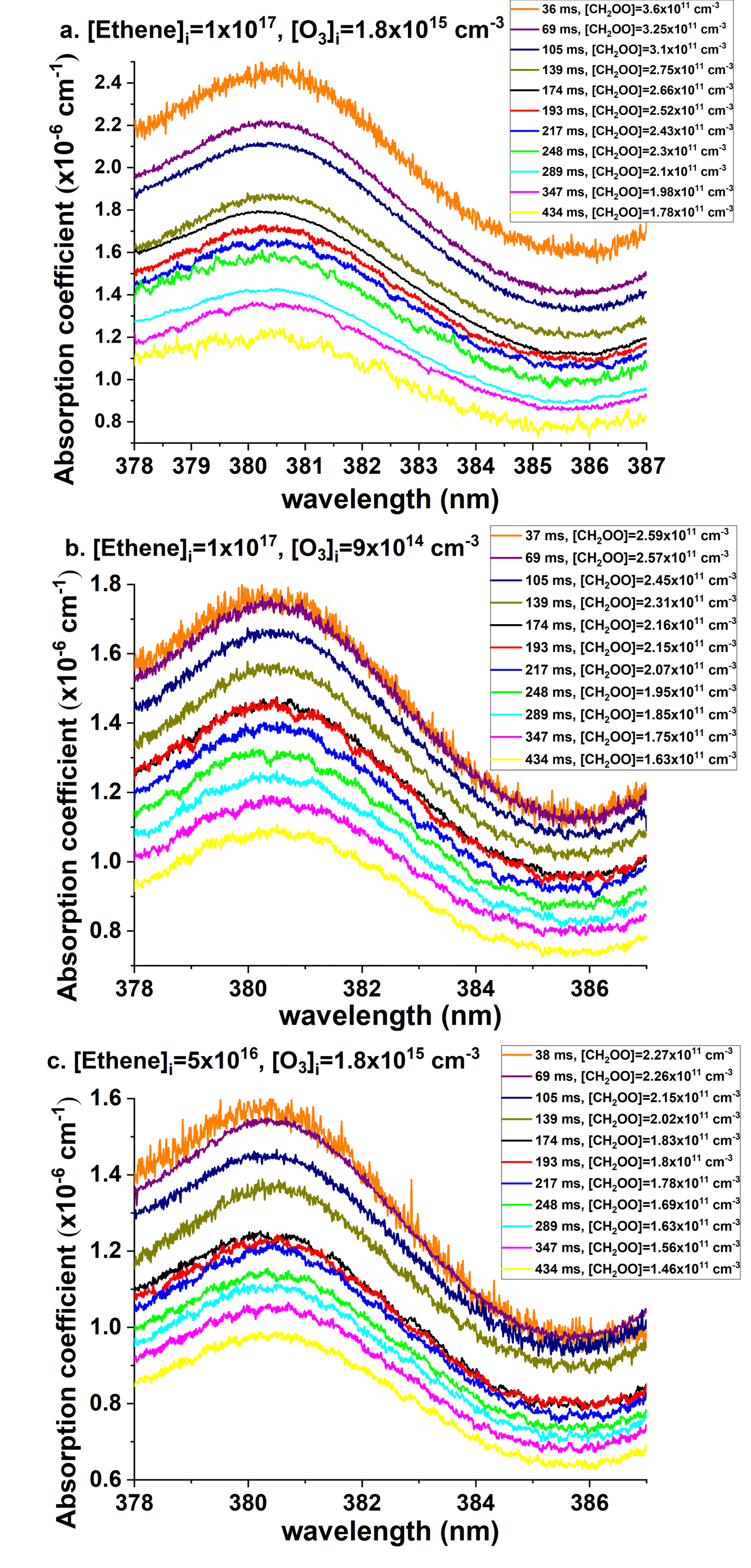
**

**Supplementary Figure 4.** **Absorption spectra of CH_2_OO** from the measured absorption coefficients of the ethene ozonolysis reactions under different residence times with the varied initial ethene and ozone concentrations of **a)** 1×10^17^ and 1.8×10^15^ cm^−3^, **b)** 1×10^17^ and 9×10^14^ cm^−3^, and **c)** 5×10^16^ and 1.8×10^15^ cm^−3^, respectively. Source data are provided as a Source Data file.


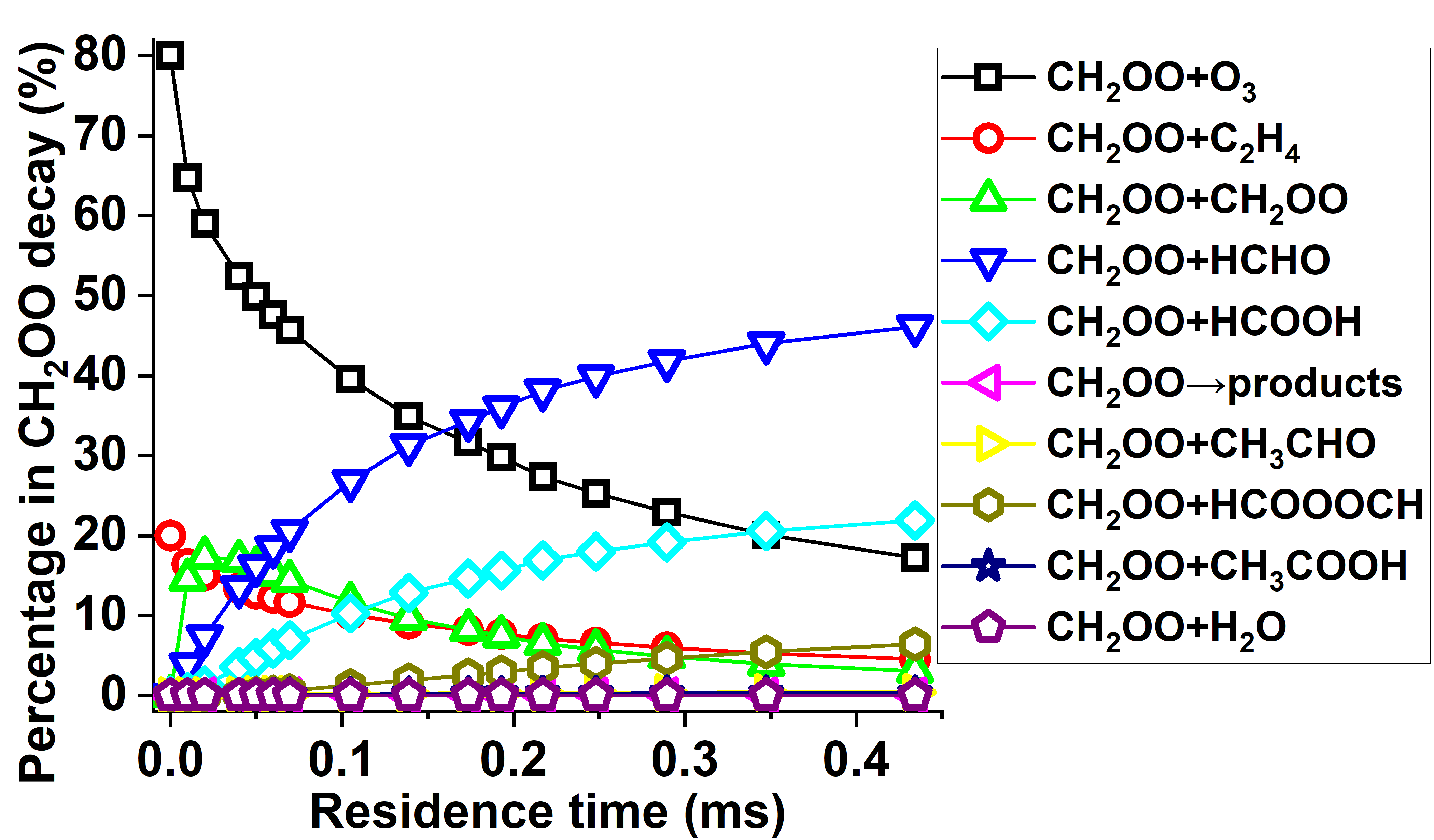


**Supplementary Figure 5.** **Percentage contributions of unimolecular and bimolecular reactions of CH_2_OO to its total decay rate** during 0 – 450 ms, calculated from the pseudo-first order reaction rates, when the initial concentrations of ethene and ozone are 1×10^17^ cm^−3^ and 1.8×10^15^ cm^−3^, respectively. Source data are provided as a Source Data file.


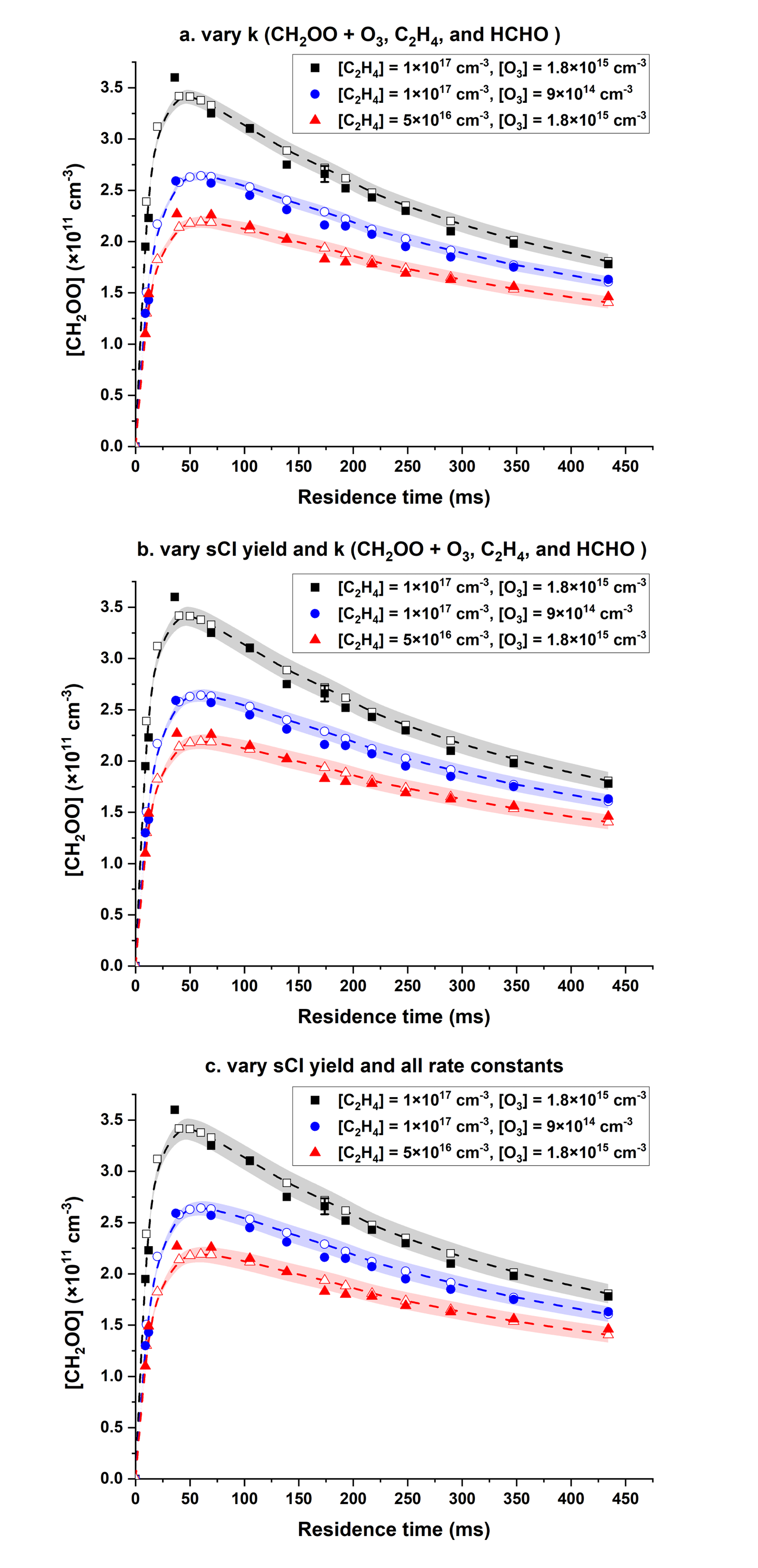


**Supplementary Figure 6.** **Concentration profile of CH_2_OO** under different residence times at 10 Torr and 293 K (solid dots: experimental data; open dots: kinetic simulation). The error bar of experimental data represents 1σ standard deviation of three repeated measurements. The error bars of kinetic simulation (colored shades) represent 1σ standard deviations **a)** when the rate constants of CH_2_OO + O_3_, C_2_H_4,_ and HCHO were randomly varied with Gaussian distribution within 4.5 (± 0.5) × 10^−14^, 2 (± 0.2) × 10^−16^ and 3.1 (± 0.3) × 10^−12^ cm^3^ s^−1^, respectively. **b)** when the sCI (stabilized CH_2_OO) yield and the rate constants of CH_2_OO + O_3_, C_2_H_4_ and HCHO were randomly varied with Gaussian distribution within 25 (± 1) %, 4.5 (± 0.5) × 10^−14^ cm^3^ s^−1^, 2 (± 0.2) × 10^−16^ cm^3^ s^−1^ and 3.1 (± 0.3) × 10^−12^ cm^3^ s^−1^, respectively. **c)** when all the rate constants in the kinetic model and the sCI yield were randomly varied with Gaussian distribution within ± 10% (relative value) and 25 (± 1) %, respectively. Source data are provided as a Source Data file.


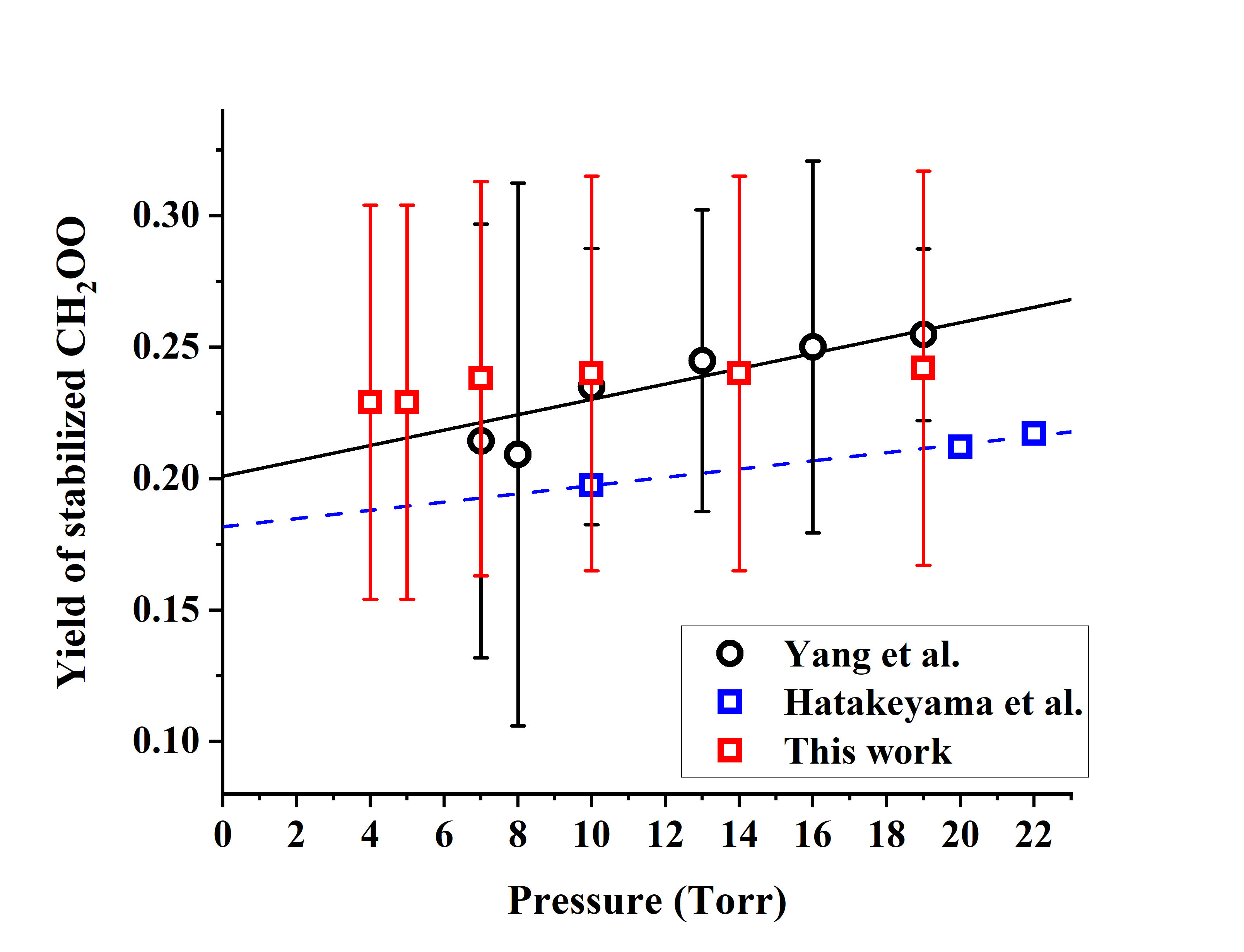


**Supplementary Figure 7.** **Yields of stabilized CH_2_OO** below 20 Torr obtained from kinetic modeling in this work and comparison with literature values^5,6^. The error bars of the black dots represent one standard deviation of three repeated measurements at each pressure^5^, while those of the red squares at each pressure in this work are estimated from the uncertainty in kinetic simulation (presented in Supplementary Figure 6) and the uncertainty of [CH_2_OO] from the CH_2_OO reference spectra. Source data are provided as a Source Data file.


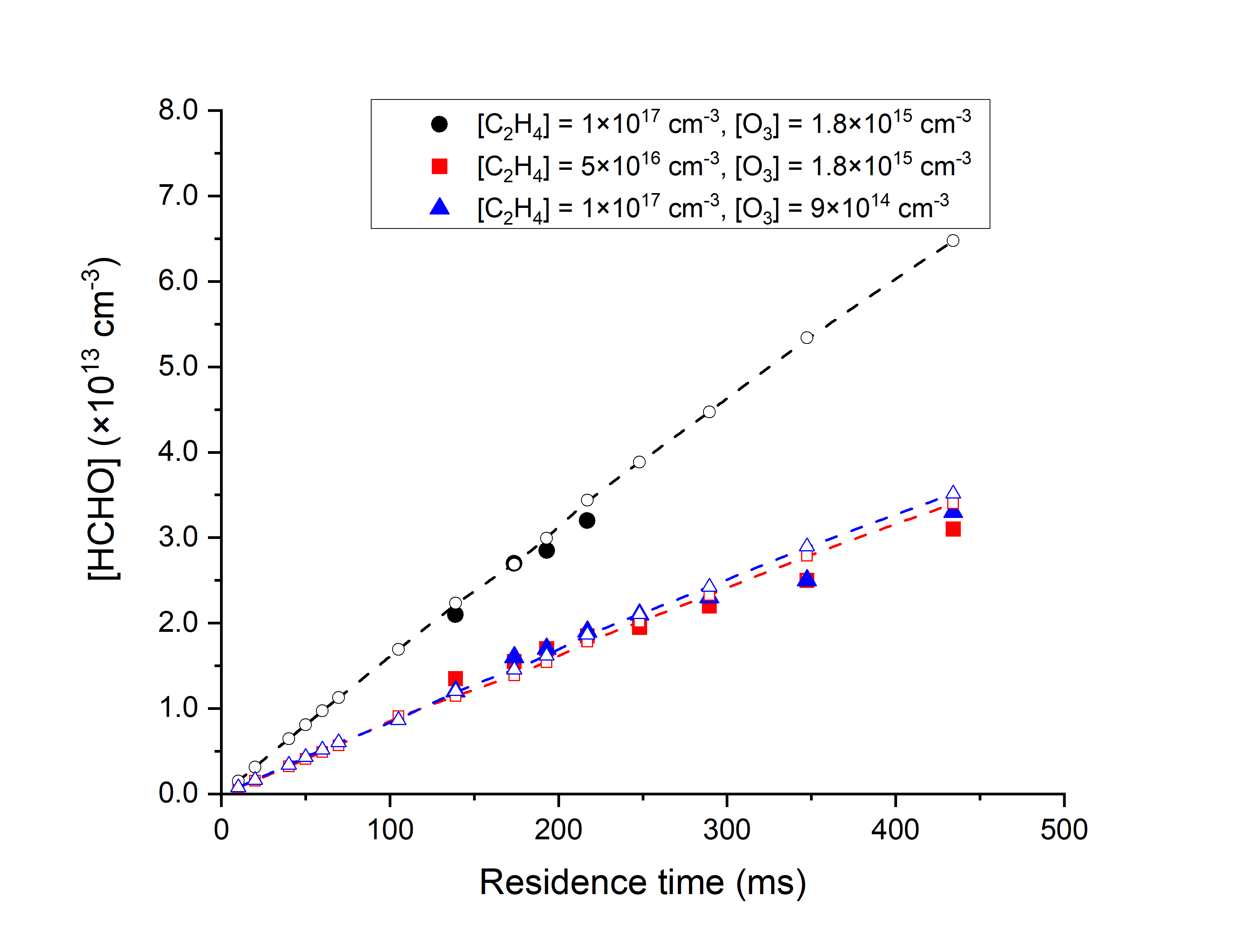


**Supplementary Figure 8.** **Concentrations of HCHO** at different residence times under different reaction conditions at 10 Torr and 293 K (solid dots: experimental data; open dots: kinetic simulation). The HCHO concentrations were obtained in the spectral range of 325 – 340 nm using high-resolution reference spectra by Smith et al^7^. Source data are provided as a Source Data file.


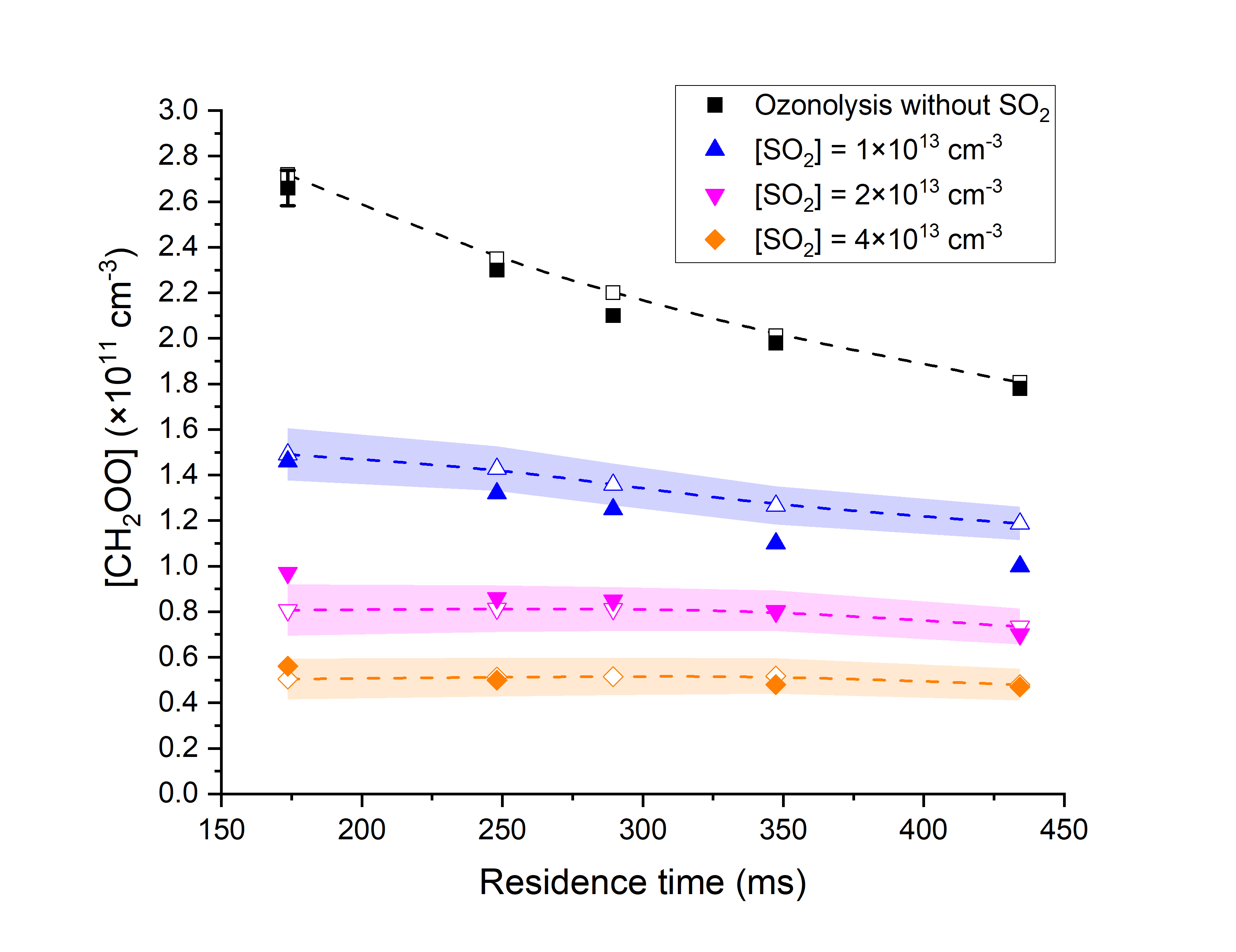


**Supplementary Figure 9.** **Concentration profile of CH_2_OO under different concentrations of SO_2_** at 10 Torr and 293 K (solid dots: experimental data; open dots: kinetic simulation). The error bar of experimental data represents 1σ standard deviation of three repeated measurements. The error bars of kinetic simulation (colored shades) represent 1σ standard deviation when the rate constant of CH_2_OO + SO_2_ was randomly varied within 3.9 (± 0.8) × 10^−11^ cm^3^ s^−1^ assuming a Gaussian distribution. Initial concentrations of ethene and ozone are 1×10^17^ cm^−3^ and 1.8×10^15^ cm^−3^, respectively. Source data are provided as a Source Data file.


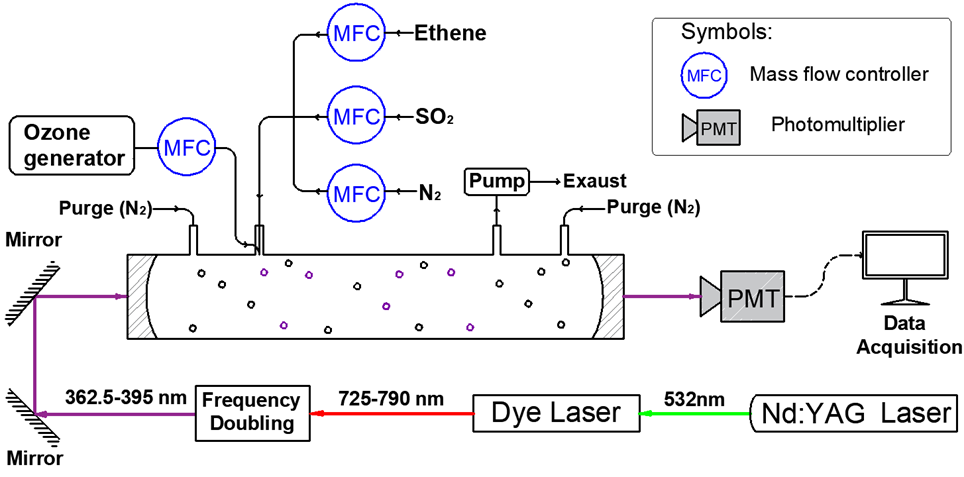


**Supplementary Figure 10.** **Experimental setup** for detection and kinetic measurements of CH_2_OO in ozonolysis of ethene. Laser intensity entering the cavity < 0.1 mJ/pulse.


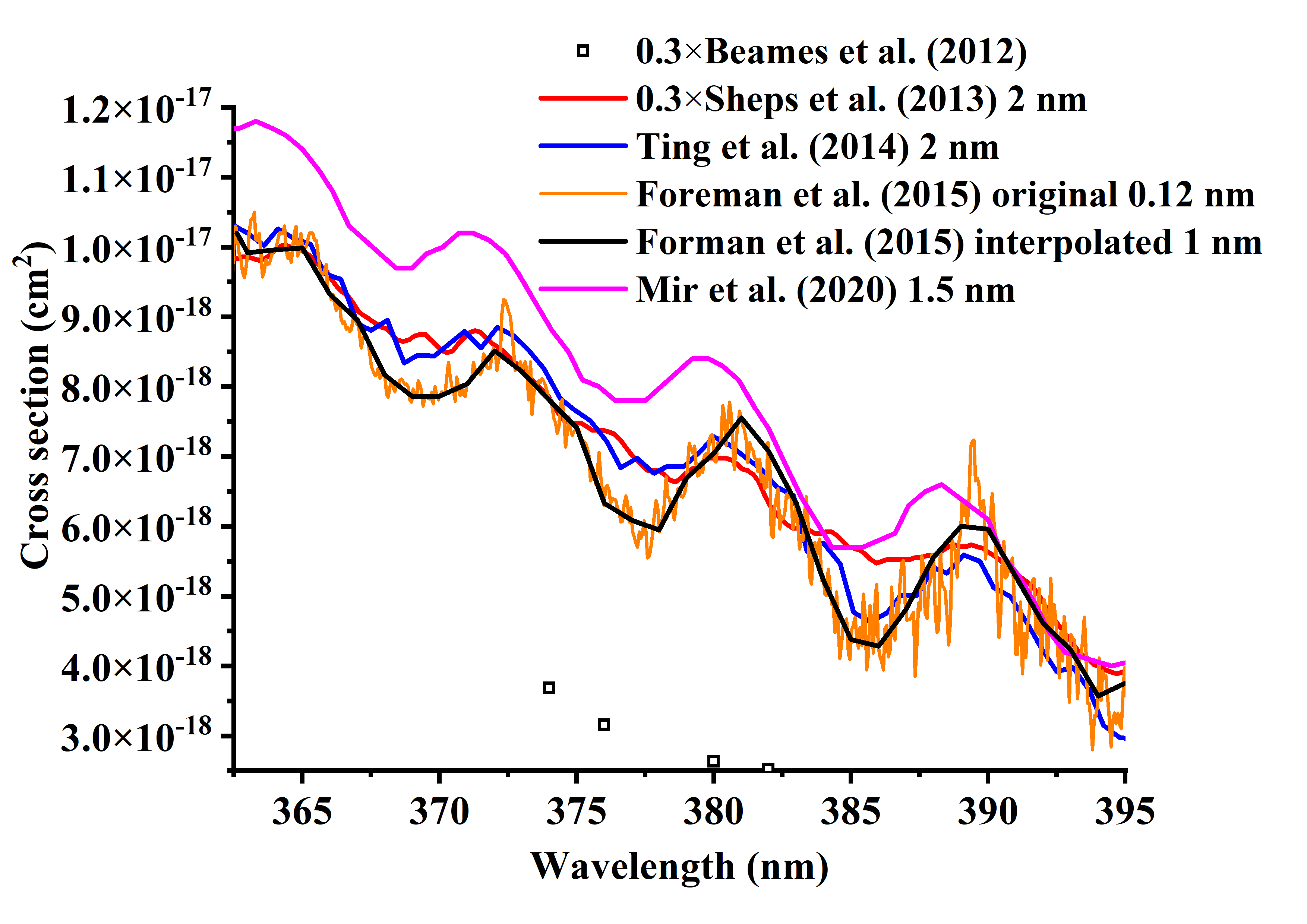


**Supplementary Figure 11.** **Available literature reference UV spectra of CH_2_OO**.^8-12^ The resolution of the reference spectra are labeled.

**Supplementary Table 1.** Flow parameters of the reactor under experimental conditions. Da and Pe are the Damkoehler and Pèclet numbers, respectively.

| Characteristic Time | Value / s | Description | |
| --- | --- | --- | --- |
| t_ck_ | 0.1 | Chemical reaction  Radial species diffusion  Radial forced convection  Axial forced convection | |
| t_sd,R_ | 0.038 |  |  |
| t_fc,R_ | 0.06 |  |  |
| t_fc,L_ | 3 |  |  |
| Negligible Axial Diffusion | | | |
| Parameter | **Value** | **Description** | **Criterion^*^** |
| (t_fc,R_)^2^/( t_sd,R_ × t_ck_) | 0.01 | Da/Pe^2^ | < 0.1 |
| t_fc,R_/ t_sd,R_ | 1.6 | Pe^−1^ | < 0.06 |
| t_ck_/ t_fc,L_ | 0.06 | Residence time v. longitudinal convection | << 1 |
| Negligible Poiseuille Flow | | | |
| t_sd,R_/ t_fc,R_ | 0.6 | Pe | < 100 |
| t_sd,R_/ t_fc,L_ | 0.012 | Radial diffusion mixing along the reactor | < 0.5 |
| t_sd,R_/ t_ck_ | 4×10^−3^ | Da | < 1 |
| t_fc,R_/ t_fc,L_ | 6×10^−3^ | Da/Pe | < 0.05 |

^*^Criteria from Cutler et al.^13^ and references therein.

**Supplementary Table 2**. Mechanism of ozonolysis of ethene used to model concentrations along the reactor. Modeling was done by dividing the PFR into CSTRs in tandem. Reference rate coefficients, yields of products, and branching ratios were summarized mainly from IUPAC^14-16^ and NIST kinetic database^17^ unless otherwise stated in the comments^18-22^. The nascent yield of thermalized CH_2_OO was set to be 23−25% to model the reaction network at 4−19 Torr. Due to limited literature information, several dummy products (as commented on) in secondary reactions were proposed to keep chemical equations balanced. For pressure-dependent reactions, the measurements closest to our experimental pressure were selected and used here. The units of rate coefficients k are s^−1^ and cm^3^ s^−1^ for unimolecular and bimolecular reactions, respectively.

| **Reaction number** | **Reaction rate coefficient (k)** | **Reactions** | **Comments** |
| --- | --- | --- | --- |
| (1) | 1.60×10^−18^ | C_2_H_4_ + O_3_ → 0.88 HCHO + 0.25 CH_2_OO + 0.63 CH_2_OO^*^ + 0.12 HOOCH_2_CHO | sCI: CH_2_OO;  hot CI: CH_2_OO^*^; Ketohydroperoxide: HOOCH_2_CHO |
|  | # | High-energy Criegee intermediate reactions |  |
| (2) | 8.00×10^3^ | CH_2_OO^*^ → CO_2_ + H_2_ | Copeland et al., 2011^18^ |
| (3) | 2.00×10^4^ | CH_2_OO^*^ → CO + H_2_O |  |
| (4) | 1.20×10^3^ | CH_2_OO^*^ → H + HCO_2_ |  |
| (5) | 3.20×10^4^ | HCO_2_ → H + CO_2_ |  |
| (6) | 7.00×10^2^ | CH_2_OO^*^ → 0.1HCOOH + 0.9HCO + 0.9OH |  |
|  | # | Secondary O_3_ reactions |  |
| (7) | 2.66×10^−11^ | H + O_3_ → OH + O_2_ |  |
| (8) | 9.13×10^−13^ | O_3_ + HOCH_2_CH_2_ → HCHO + CH_2_OH + O_2_ |  |
| (9) | 9.13×10^−13^ | O_3_ + CH_2_OH → HCHO + OH + O_2_ |  |
| (10) | 7.30×10^−14^ | OH + O_3_ → HO_2_ + O_2_ |  |
| (11) | 2.00×10^−15^ | HO_2_ + O_3_ → OH + 2O_2_ |  |
| (12) | 8.00×10^−15^ | O + O_3_ → 2O_2_ |  |
|  | # | Secondary C_2_H_4_ Reactions |  |
| (13) | 3.82×10^−13^ | C_2_H_4_ + H → C_2_H_5_ |  |
| (14) | 1.50×10^−13^ | C_2_H_5_ + O_2_ → C_2_H_4_ + HO_2_ |  |
| (15) | 1.70×10^−11^ | C_2_H_5_ + O_2_ → C_2_H_5_O_2_ | Pressure dependent |
| (16) | 4.80×10^−14^ | C_2_H_5_O_2_ + C_2_H_5_O_2_ → 2C_2_H_5_O + O_2_ |  |
| (17) | 2.80×10^−14^ | C_2_H_5_O_2_ + C_2_H_5_O_2_ → C_2_H_5_OH + CH_3_CHO + O_2_ |  |
| (18) | 1.00×10^−16^ | C_2_H_5_O_2_ + C_2_H_5_O_2_ → C_2_H_5_OOC_2_H_5_ + O_2_ |  |
| (19) | 3.80×10^−12^ | C_2_H_4_ + OH → HOCH_2_CH_2_ |  |
|  | # | stabilized Criegee intermediate reactions |  |
| (20) | 4.50×10^−14^ | CH_2_OO + O_3_ → HCHO + O_2_ + O_2_ | Major consumption reactions of CH_2_OO. Rate constants from fitting to experimental data of this work (Fig. 3) |
| (21) | 2.00×10^−16^ | CH_2_OO + C_2_H_4_ → HCHO + CH_3_CHO |  |
| (22) | 1.80×10^−12^ | CH_2_OO + HCHO → HCOOH + HCHO |  |
| (23) | 4.40×10^−13^ | CH_2_OO + HCHO → CO + H_2_O + HCHO |  |
| (24) | 4.40×10^−13^ | CH_2_OO + HCHO → HCO + OH + HCO + H |  |
| (25) | 4.40×10^−13^ | CH_2_OO + HCHO → CH_3_CHO + O_2_ |  |
| (26) | 0.1 | CH_2_OO → 0.7HCHO_2_ + 0.3HCOOH |  |
| (27) | 0.1 | CH_2_OO → HHOOC | Wall dummy |
| (28) | 2.41×10^−16^ | CH_2_OO + H_2_O → CH_4_O_3_ | Water dummy |
| (29) | 7.40×10^−11^ | CH_2_OO + CH_2_OO → 2HCHO + O_2_ |  |
| (30) | 1.10×10^−10^ | CH_2_OO + HCOOH → HCOOOCH + H_2_O |  |
| (31) | 9.50×10^−13^ | CH_2_OO + CH_3_CHO → HCHO + CH_3_COOH | Taatjes et al., 2012^21^ |
| (32) | 1.30×10^−10^ | CH_2_OO + CH_3_COOH → C_3_H_6_O_4_ | Dummy product |
| (33) | 1.00×10^−11^ | CH_2_OO + HCOOOCH → H_4_C_3_O_5_ | Dummy product |
| (34) | 3.90×10^−11^ | CH_2_OO + SO_2_ → HCHO + SO_3_ | The rate constant from fitting to experimental data (Supplementary Fig. 9) |
|  | # | HO_x_ reactions (non-VOC) |  |
| (35) | 5.60×10^−12^ | H + HO_2_ → H_2_ + O_2_ |  |
| (36) | 7.20×10^−11^ | H + HO_2_ → 2OH |  |
| (37) | 2.40×10^−12^ | H + HO_2_ → H_2_O + O |  |
| (38) | 3.50×10^−11^ | O + OH → O_2_ + H |  |
| (39) | 5.80×10^−11^ | O + HO_2_ → OH + O_2_ |  |
| (40) | 1.70×10^−15^ | O + H_2_O_2_ → OH + HO_2_ |  |
| (41) | 6.70×10^−15^ | H_2_ + OH → H_2_O + H |  |
| (42) | 1.48×10^−12^ | OH + OH → H_2_O + O |  |
| (43) | 2.60×10^−13^ | OH + OH → H_2_O_2_ | Pressure dependent |
| (44) | 1.10×10^−10^ | OH + HO_2_ → H_2_O + O_2_ |  |
| (45) | 1.70×10^−12^ | OH + H_2_O_2_ → H_2_O + HO_2_ |  |
| (46) | 1.60×10^−12^ | HO_2_ + HO_2_ → H_2_O_2_ + O_2_ | Pressure-dependent |
|  | # | Secondary O_2_ Reactions |  |
| (47) | 1.27×10^−14^ | H + O_2_ → HO_2_ | Pressure dependent |
| (48) | 1.80×10^−16^ | O + O_2_ → O_3_ | Pressure dependent |
| (49) | 5.20×10^−12^ | HCO + O_2_ → CO + HO_2_ |  |
| (50) | 5.10×10^−12^ | CH_3_CO + O_2_ → CH_3_COOO | Pressure dependent |
|  | # | Secondary CH_2_CHO (vinoxy) + O_2_ reactions |  |
| (51) | 1.60×10^−14^ | CH_2_CHO + O_2_ → CH_2_CO + HO_2_ |  |
| (52) | 6.00×10^−15^ | CH_2_CHO + O_2_ → CHOCHO + OH |  |
| (53) | 8.00×10^−15^ | CH_2_CHO + O_2_ → HCHO + CO + OH |  |
| (54) | 6.00×10^−14^ | CH_2_CHO + O_2_ → OOCH_2_CHO | Dummy product |
|  | # | HO_x_ reactions with VOCs |  |
| (55) | 1.44×10^−13^ | OH + CO → H + CO_2_ |  |
| (56) | 8.50×10^−12^ | OH + HCHO → H_2_O + HCO |  |
| (57) | 1.43×10^−11^ | OH + CH_3_CHO → H_2_O + CH_3_CO |  |
| (58) | 7.50×10^−13^ | OH + CH_3_CHO → H_2_O + CH_2_CHO |  |
| (59) | 4.50×10^−13^ | OH + HCOOH → CH_3_O_3_ | Dummy product |
| (60) | 7.90×10^−14^ | HO_2_ + HCHO → HOCH_2_OO |  |
| (61) | 1.50×10^2^ | HOCH_2_OO → HO_2_ + HCHO |  |
| (62) | 7.00×10^−13^ | HOCH_2_OO + HOCH_2_OO → HCOOH + CH_2_OHOH + O_2_ |  |
| (63) | 5.50×10^−12^ | HOCH_2_OO + HOCH_2_OO → 2HOCH_2_O + O_2_ |  |
| (64) | 6.00×10^−12^ | HO_2_ + HOCH_2_OO → O_2_ + HOCH_2_O_2_H |  |
| (65) | 4.00×10^−12^ | HO_2_ + HOCH_2_OO → O_2_ + HCOOH + H_2_O |  |
| (66) | 2.00×10^−12^ | HO_2_ + HOCH_2_OO → O_2_ + OH + HOCH_2_O |  |
|  | # | HOCH_2_CH_2_ + O_2_ Pathways |  |
| (67) | 3.00×10^−12^ | HOCH_2_CH_2_ + O_2_ → HOCH_2_CH_2_O_2_ |  |
| (68) | 1.00×10^−9^ | HOCH_2_CH_2_O_2_ → HO_2_ + CH_2_CHOH |  |
| (69) | 1.00×10^−9^ | HOCH_2_CH_2_O_2_ → CH_2_OH + CH_2_OO | Criegee intermediate formation |
| (70) | 1.10×10^−12^ | HOCH_2_CH_2_O_2_ + HOCH_2_CH_2_O_2_ → HOCH_2_CH_2_OH + HOCH_2_CHO + O_2_ |  |
| (71) | 1.10×10^−12^ | HOCH_2_CH_2_O_2_ + HOCH_2_CH_2_O_2_ → 2HOCH_2_CH_2_O + O_2_ |  |
| (72) | 1.30×10^−13^ | HOCH_2_CH_2_O_2_ + HO_2_ → HOCH_2_CH_2_OOH + O_2_ | Dummy ratio |
| (73) | 1.30×10^−11^ | HOCH_2_CH_2_O_2_ + HO_2_ → HOCH_2_CHO + H_2_O + O_2_ |  |
| (74) | 6.40×10^−12^ | OH + HOCH_2_CHO → H_2_O + HOCH_2_CO |  |
| (75) | 1.60×10^−12^ | OH + HOCH_2_CHO → H_2_O + HOCHCHO |  |
| (76) | 1.45×10^−11^ | OH + HOCH_2_CH_2_OH → C_2_H_7_O_3_ | Dummy product |
|  | # | HOCH_2_CH_2_ + O_2_ pathways (unknown) |  |
| (77) | 1.00×10^−16^ | HOCH_2_CH_2_O + O_2_ → HOCH_2_CHO + HO_2_ | Rate at low pressure (guess) |
| (78) | 10 | HOCH_2_CH_2_O → CH_2_OH + HCHO |  |
| (79) | 9.70×10^−12^ | CH_2_OH + O_2_ → HCHO + HO_2_ |  |
|  |  | KHP |  |
| (80) | 2.5 | HOOCH_2_CHO → HCHO+HCO+OH |  |
| (81) | 2.5 | HOOCH2CHO → CHOCHO+OH+H |  |
| (82) | 2.5 | HOOCH_2_CHO → CHOCHO+H_2_O |  |

**Supplementary Table 3.** Pseudo-first order reaction rates of unimolecular and bimolecular reactions of CH_2_OO at the beginning and end of the time frame studied in this work when initial concentrations of ethene and ozone are 1×10^17^ cm^−3^ and 1.8×10^15^ cm^−3^, respectively. The corresponding reaction numbers in Supplementary Table 2 are listed for cross-references.

| Rate constant k (cm^3^ s^−1^) | | Reaction  CH_2_OO + X → products | Reaction number in Supplementary Table 2 | Average concentration of X (cm^−3^) from the kinetic model | | Pseudo-first-order reaction rates k[X] (s^−1^) | |
| --- | --- | --- | --- | --- | --- | --- | --- |
| Residence time (ms) | | |  | 10 ms | 434 ms | 10 ms | 434 ms |
| 4.50×10^−14^ | | CH_2_OO + O_3_ →  HCHO + 2O_2_ | R20 | 1.75×10^15^ | 1.68×10^15^ | 79 | 76 |
| 2.00×10^−16^ | | CH_2_OO + C_2_H_4_ → HCHO + CH_3_CHO | R21 | 1.00×10^17^ | 9.99×10^16^ | 20 | 20 |
| 7.40×10^−11^ | | CH_2_OO + CH_2_OO → 2HCHO + O_2_ | R29 | 2.39×10^11^ | 1.81×10^11^ | 35 | 27 |
| 3.12×10^−12^ | | CH_2_OO + HCHO → HCOOH + HCHO or other products | R22 – R25^*^ | 1.50×10^12^ | 6.48×10^13^ | 5 | 202 |
| 1.10×10^−10^ | | CH_2_OO + HCOOH → HCOOOCH + H_2_O | R30 | 4.47×10^9^ | 8.73×10^11^ | 0.5 | 96 |
| 0.20 s^−1^ | | CH_2_OO → products | R26, R27 | N/A | N/A | 0.2 | 0.2 |
| 9.50×10^−13^ | | CH_2_OO + CH_3_CHO → HCHO + CH_3_COOH | R31 | 2.17×10^10^ | 1.87×10^12^ | 0.02 | 2 |
| 1.00×10^−11^ | | CH_2_OO+HCOOOCH → products | R33 | 4.47×10^8^ | 2.81×10^12^ | 0.004 | 28 |
| 1.30×10^−10^ | | CH_2_OO+CH_3_COOH → products | R32 | 1.72×10^7^ | 1.13×10^10^ | 0.002 | 1 |
| 2.4×10^−16^ | | CH_2_OO+H_2_O → products | R28 | 6.48×10^11^ | 3.59×10^13^ | 0.0002 | 0.01 |
|  | Total reaction rate | | | | | 139 | 452 |

^*^ The reaction of CH_2_OO + HCHO → HCOOH + HCHO takes up to 58 % among the four reaction pathways of CH_2_OO + HCHO in our model (see R22 – R25 in Supplementary Table 2).

**Supplementary references**

1. Bogumil, K. *et al.* Measurements of Molecular Absorption Spectra with the SCIAMACHY Pre-Flight Model: Instrument Characterization and Reference Data for Atmospheric Remote-Sensing in the 230–2380 nm Region. *J. Photochem. Photobiol., A* **157**, 167-184 (2003).

2. Robinson, G. W. & DiGiorgio, V. E. The Nature of Formaldehyde in its Low-Lying Excited States. *Can. J. Chem.* **36**, 31-38 (1958).

3. Vandaele, A. C., Hermans, C. & Fally, S. Fourier Transform Measurements of SO_2_ Absorption Cross Sections: II. Temperature Dependence in the 29000–44000cm^−1^ (227–345nm) Region. *J. Quant. Spectrosc. Radiat. Transf.* **110**, 2115-2126 (2009).

4. Keller-Rudek, H., Moortgat, G. K., Sander, R. & Sorensen, R. The MPI-Mainz UV/VIS Spectral Atlas of Gaseous Molecules of Atmospheric Interest. *Earth Syst. Sci. Data* **5**, 365-373 (2013).

5. Yang, L., Campos-Pineda, M. & Zhang, J. Low-Pressure and Nascent Yields of Thermalized Criegee Intermediate in Ozonolysis of Ethene. *J. Phys. Chem. Lett.* **13**, 11496-11502 (2022).

6. Hatakeyama, S., Kobayashi, H., Lin, Z. Y., Takagi, H. & Akimoto, H. Mechanism for the Reaction of CH_2_OO with SO_2_. *J. Phys. Chem.* **90**, 4131-4135 (1986).

7. Smith, C. A., Pope, F. D., Cronin, B., Parkes, C. B. & Orr-Ewing, A. J. Absorption Cross Sections of Formaldehyde at Wavelengths from 300 to 340 nm at 294 and 245 K. *J. Phys. Chem. A* **110**, 11645-11653 (2006).

8. Ting, W.-L., Chen, Y.-H., Chao, W., Smith, M. C. & Lin, J. J.-M. The UV Absorption Spectrum of the Simplest Criegee Intermediate CH_2_OO. *Phys. Chem. Chem. Phys.* **16**, 10438-10443 (2014).

9. Foreman, E. S. *et al.* High Resolution Absolute Absorption Cross Sections of the B^1^A'-X^1^A' Transition of the CH_2_OO Biradical. *Phys. Chem. Chem. Phys.* **17**, 32539-32546 (2015).

10. Mir, Z. S. *et al.* CH_2_OO Criegee intermediate UV absorption cross-sections and kinetics of CH_2_OO + CH_2_OO and CH_2_OO + I as a function of pressure. *Phys. Chem. Chem. Phys.* **22**, 9448-9459 (2020).

11. Beames, J. M., Liu, F., Lu, L. & Lester, M. I. Ultraviolet Spectrum and Photochemistry of the Simplest Criegee Intermediate CH_2_OO. *J. Am. Chem. Soc.* **134**, 20045-20048 (2012).

12. Sheps, L. Absolute Ultraviolet Absorption Spectrum of a Criegee Intermediate CH_2_OO. *J. Phys. Chem. Lett.* **4**, 4201-4205 (2013).

13. Cutler, A. H., Antal, M. J. & Jones, M. A Critical Evaluation of the Plug-Flow Idealization of Tubular-Flow Reactor Data. *Ind. Eng. Chem. Res.* **27**, 691-697 (1988).

14. Cox, R. A. *et al.* Evaluated Kinetic and Photochemical Data for Atmospheric Chemistry: Volume VII – Criegee intermediates. *Atmos. Chem. Phys.* **20**, 13497-13519 (2020).

15. Atkinson, R. *et al.* Evaluated Kinetic and Photochemical Data for Atmospheric Chemistry: Volume II – Gas Phase Reactions of Organic Species. *Atmos. Chem. Phys.* **6**, 3625-4055 (2006).

16. Atkinson, R. *et al.* Evaluated Kinetic and Photochemical Data for Atmospheric Chemistry: Volume I - Gas Phase Reactions of O_x_, HO_x_, NO_x_ and SO_x_ species. *Atmos. Chem. Phys.* **4**, 1461-1738 (2004).

17. Manion, J. A. *et al.* *NIST Chemical Kinetics Database, NIST Standard Reference Database 17, Version 7.0 (Web Version), Release 1.6.8, Data version 2015.09*, <<https://kinetics.nist.gov/>> (2023).

18. Copeland, G., Ghosh, M. V., Shallcross, D. E., Percival, C. J. & Dyke, J. M. A Study of the Ethene-Ozone Reaction with Photoelectron Spectroscopy: Measurement of Product Branching Ratios and Atmospheric Implications. *Phys. Chem. Chem. Phys.* **13**, 14839 (2011).

19. Chhantyal-Pun, R., Davey, A., Shallcross, D. E., Percival, C. J. & Orr-Ewing, A. J. A Kinetic Study of the CH_2_OO Criegee Intermediate Self-Reaction, Reaction with SO_2_ and Unimolecular Reaction using Cavity Ring-Down Spectroscopy. *Phys. Chem. Chem. Phys.* **17**, 3617-3626 (2015).

20. Welz, O. *et al.* Rate Coefficients of C_1_ and C_2_ Criegee Intermediate Reactions with Formic and Acetic Acid Near the Collision Limit: Direct Kinetics Measurements and Atmospheric Implications. *Angew. Chem., Int. Ed.* **53**, 4547-4550 (2014).

21. Taatjes, C. A. *et al.* Direct Measurement of Criegee Intermediate (CH_2_OO) Reactions with Acetone, Acetaldehyde, and Hexafluoroacetone. *Phys. Chem. Chem. Phys.* **14**, 10391-10400 (2012).

22. Buras, Z. J., Elsamra, R. M. I., Jalan, A., Middaugh, J. E. & Green, W. H. Direct Kinetic Measurements of Reactions between the Simplest Criegee Intermediate CH_2_OO and Alkenes. *J. Phys. Chem. A* **118**, 1997-2006 (2014).
